# 

**Published:** 1888-12-22

**Authors:** 


PERMANENTLY ENLARGED TO 32 PAGES.

PRICE ONE PENNY.

No. 117, Vol. V.—December 22nd, 1888.

[Registered at the General Post Office as a Newspaper.]

Per Annum, 6s. 6d., post free.
Monthly Parts. 6d.; post free, 7½d.
Ditto per Ann. 7s. 6d., post free.

### THE HOSPITAL

A Weekly Institutional Journal of

